# Supplementary material for: Multiple random phosphorylations in clock proteins provide long delays and switches
Source: Sci Rep. 2020 Dec 17;10:22224. doi: 10.1038/s41598-020-79277-z (PMC7746754; doi:10.1038/s41598-020-79277-z)
Supplement: Supplementary file 1 — Supplementary information. [file 41598_2020_79277_MOESM1_ESM.pdf]

Supplementary Material

Multiple random phosphorylations in clock proteins provide long delays and switches

Abhishek Upadhyay [1*], Daniela Marzoll [2], Axel Diernfellner [2], Michael Brunner [2] and Hanspeter Herzel [1*]

[1] Institute for Theoretical Biology, Charité – Universitätsmedizin Berlin and Humboldt University of Berlin, Philippstr. 13, 10115, Berlin, Germany; abhishek.upadhyay@charite.de, hans-peter.herzel@charite.de
[2] Biochemistry Center, University of Heidelberg, ImNeuenheimer Feld 328, 69120, Heidelberg, Germany; daniela.marzoll@bzh.uni-heidelberg.de, axel.diernfellner@bzh.uni-heidelberg.de, michael.brunner@bzh.uni-heidelberg.de
* Correspondence: abhibiotechnologist@gmail.com, h.herzel@biologie.hu-berlin.de; Tel.: +4930 2093 98402

Appendix (Supporting Information)

Appendix A.1 Scheme of the linear model, equations and parameters

We modelled the linear phosphorylations using five-variable ordinary differential equations (ODEs). Mass-action kinetics of processive phosphorylations provide long delays shown in Fig 2 with the parameters P_p=50, k=50h^{-1} and k_d=0.1h^{-1}.

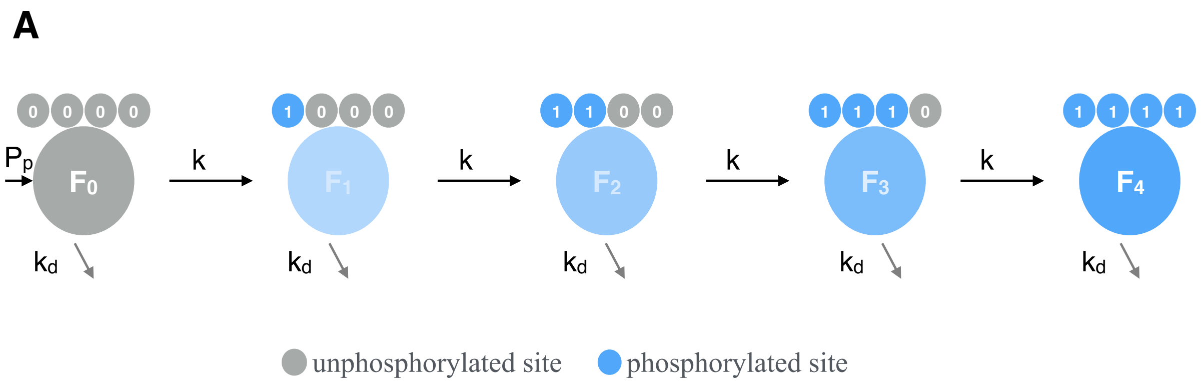

B

$$\frac{d[F_0]}{dt} = P_p - k * [F_0] - k_d * [F_0]$$

$$\frac{d[F_1]}{dt} = k * [F_0] - k * [F_1] - k_d * [F_1]$$

$$\frac{d[F_2]}{dt} = k * [F_1] - k * [F_2] - k_d * [F_2]$$

$$\frac{d[F_3]}{dt} = k * [F_2] - k * [F_3] - k_d * [F_3]$$

$$\frac{d[F_4]}{dt} = k * [F_3] - k_d * [F_4]$$

Figure A1. (A) Linear model: Schematic diagram of phosphorylations with turnover of protein F: F is phosphorylated in a sequential processive manner. **(B) Model of up to 4 phosphorylations with 5 variables.**

Appendix A.2 Scheme of the nonlinear model, equations and parameters

We modelled the nonlinear phosphorylations using a ten-variable ODE system. Distributive kinetics of phosphorylations enhances ultrasensitivity shown in Fig 3. Parameters in Figs 3 and 5: k_1=50, k_2=50h^{-1}, k_d=0.1h^{-1}, P_p=500 and P_c=1. Fig 4: P_c=5.

A

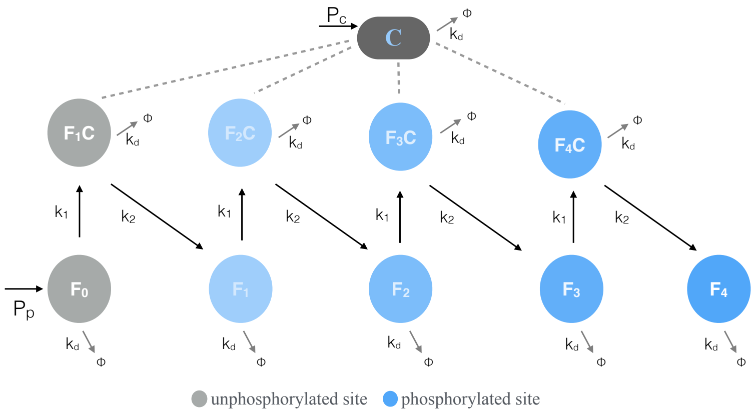

B

$$\frac{d[F_0]}{dt} = P_p - k_1 * [F_0] * [C] - k_d * [F_0]$$
$$\frac{d[F_1C]}{dt} = k_1 * [F_0] * [C] - k_2 * [F_1C] - k_d * [F_1C]$$
$$\frac{d[F_1]}{dt} = k_2 * [F_1C] - k_1 * [F_1] * [C] - k_d * [F_1]$$
$$\frac{d[F_2C]}{dt} = k_1 * [F_1] * [C] - k_2 * [F_2C] - k_d * [F_2C]$$
$$\frac{d[F_2]}{dt} = k_2 * [F_2C] - k_1 * [F_2] * [C] - k_d * [F_2]$$
$$\frac{d[F_3C]}{dt} = k_1 * [F_2] * [C] - k_2 * [F_3C] - k_d * [F_3C]$$
$$\frac{d[F_3]}{dt} = k_2 * [F_3C] - k_1 * [F_3] * [C] - k_d * [F_3]$$
$$\frac{d[F_4C]}{dt} = k_1 * [F_3] * [C] - k_2 * [F_4C] - k_d * [F_4C]$$
$$\frac{d[F_4]}{dt} = k_2 * [F_4C] - k_d * [F_4]$$

$$\frac{d[C]}{dt} = P_c - k_d * [C] + (k_2 * [F_1C] + k_2 * [F_2C] + k_2 * [F_3C] + k_2 * [F_4C]) - (k_1 * [F_0] * [C] + k_1 * [F_1] * [C] + k_1 * [F_2] * [C] + k_1 * [F_3] * [C])$$

Figure A2. (A) Nonlinear model: Schematic diagram of protein phosphorylations with turnover of F and C: F is phosphorylated by C in a sequential distributive manner. **(B) Model of up to 4 phosphorylations of F with 10 variables.**

Appendix A.3 Noninear random model: scheme, equations and parameters

We modelled the nonlinear random phosphorylations using a ten-variable ODE system. Distributive kinetics and prefactors associated to random phosphorylations together provide large amplitudes of phosphorylations shown in Fig 6. Parameters in Fig 6: k_1=50, k_2=50h^{-1}, k_d=0.1h^{-1}, P_p=500 and P_c=1.

A

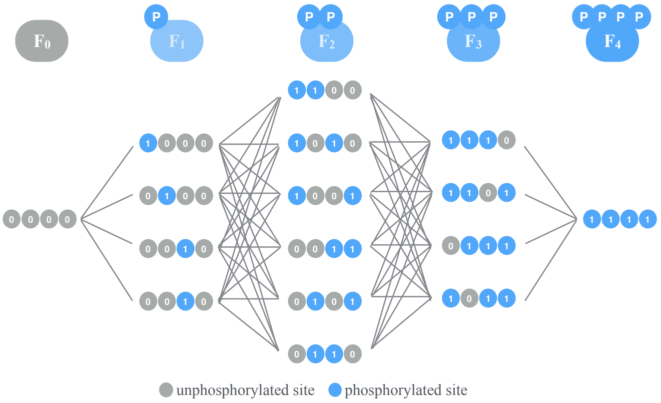

B

$$\frac{d[F_0]}{dt} = P_p - k_1 * [F_0] * [C] - k_d * [F_0]$$
$$\frac{d[F_1C]}{dt} = k_1 * [F_0] * [C] - k_2 * [F_1C] - k_d * [F_1C]$$
$$\frac{d[F_1]}{dt} = 4 * k_2 * [F_1C] - k_1 * [F_1] * [C] - k_d * [F_1]$$
$$\frac{d[F_2C]}{dt} = k_1 * [F_1] * [C] - k_2 * [F_2C] - k_d * [F_2C]$$
$$\frac{d[F_2]}{dt} = 3 * k_2 * [F_2C] - k_1 * [F_2] * [C] - k_d * [F_2]$$
$$\frac{d[F_3C]}{dt} = k_1 * [F_2] * [C] - k_2 * [F_3C] - k_d * [F_3C]$$
$$\frac{d[F_3]}{dt} = 2 * k_2 * [F_3C] - k_1 * [F_3] * [C] - k_d * [F_3]$$
$$\frac{d[F_4C]}{dt} = k_1 * [F_3] * [C] - k_2 * [F_4C] - k_d * [F_4C]$$
$$\frac{d[F_4]}{dt} = 1 * k_2 * [F_4C] - k_d * [F_4]$$

$$\frac{d[C]}{dt} = P_c - k_d * [C] + (k_2 * [F_1C] + k_2 * [F_2C] + k_2 * [F_3C] + k_2 * [F_4C]) - (k_1 * [F_0] * [C] + k_1 * [F_1] * [C] + k_1 * [F_2] * [C] + k_1 * [F_3] * [C])$$

Figure A3. (A) Nonlinear random model: Schematic diagram of phosphorylations with turnover of protein F and kinase C: F is phosphorylated by C in a distributive random manner. **(B) Model of up to n=4 phosphorylations.**

Appendix A.4 Large scale nonlinear random model: scheme, equations and parameters

We modelled the large scale nonlinear random phosphorylations using a 2n+2 (n=100) variable ODE system. Distributive kinetics, prefactors associated to random phosphorylations and longer chain of phosphorylations are shown in Fig 7. Amplitudes, delays and Hill coefficients for 100 phosphorylations shown in Fig 8 are also derived from model simulations. Parameters in Fig 7: k_1=50000, k_2=50000h^{-1}, k_d=1.5h^{-1}, P_p=0.01 and P_c=10. Default (blue) parameters in Fig 8: k_1=50000, k_2=50000h^{-1}, k_d=5h^{-1}, P_p=1 and P_c=10. Slow degradation (red): k_d=0.15h^{-1} and high kinase/protein ratio (green): P_c=100.

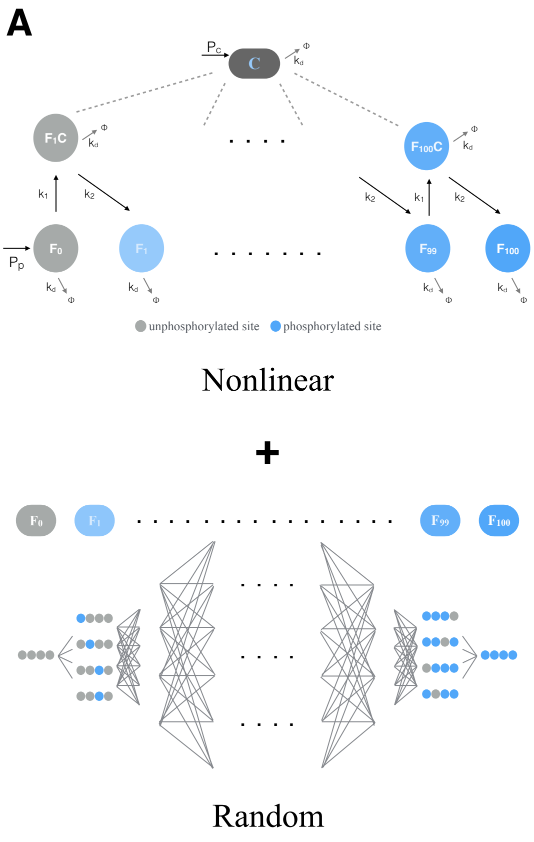

B

$$\frac{d[F_0]}{dt} = P_p - k_1 * [F_0] * [C] - k_d * [F_0]$$
$$\frac{d[F_1C]}{dt} = k_1 * [F_0] * [C] - k_2 * [F_1C] - k_d * [F_1C]$$
$$\frac{d[F_1]}{dt} = 100 * k_2 * [F_1C] - k_1 * [F_1] * [C] - k_d * [F_1]$$

.
.
.
.

$$\frac{d[F_{99}]}{dt} = 2 * k_2 * [F_{99}C] - k_1 * [F_{99}] * [C] - k_d * [F_{99}]$$
$$\frac{d[F_{100}C]}{dt} = k_1 * [F_{99}] * [C] - k_2 * [F_{100}C] - k_d * [F_{100}C]$$
$$\frac{d[F_{100}]}{dt} = 1 * k_2 * [F_{100}C] - k_d * [F_{100}]$$

$$\frac{d[C]}{dt} = P_c - k_d * [C] + (k_2 * [F_1C] + ... + k_2 * [F_{99}C] + k_2 * [F_{100}C]) - (k_1 * [F_0] * [C] + k_1 * [F_1] * [C] + ... + k_1 * [F_{99}] * [C])$$

Figure A4. (A) Large scale nonlinear random model: Schematic diagram of phosphorylations with turnover of protein F and kinase C: F is phosphorylated by C in a distributive random manner. **(B) Model of up to n=100 phosphorylations with 2n+2 variables.**

Appendix A.5 Hill coefficients and delays

Hill coefficients and delays are calculated using eqns 1, 2 and 3 for the curves shown in Figs 2, 3 and 6.

Protein phospho- -forms	Linear (Figure 2)		Nonlinear (Figure 3)		Nonlinear random (Figure 6)	
	Hill coefficient	*Delay*	*Hill coefficient*	*Delay*	*Hill coefficient*	*Delay*
**F0**	*1.18*	*2.6*	*1.77*	*1.7*	*1.66*	*2.8*
**F1**	*1.61*	*6.4*	*2.45*	*4.2*	*2.19*	*4.6*
**F2**	*1.99*	*10.4*	*2.98*	*6.8*	*2.60*	*6.8*
**F3**	*2.78*	*14.4*	*3.43*	*9.5*	*2.93*	*9.4*
**F4**	*3.39*	*18.4*	*3.85*	*14.7*	*3.20*	*13.3*

Figure A5. Hill coefficients and delays: A list of Hill coefficients and time delays of the phosphorylated Fk across three models. See Figs 2, 3 and 6.

Appendix A.6 Enlarged time series of nonlinear phosphorylations

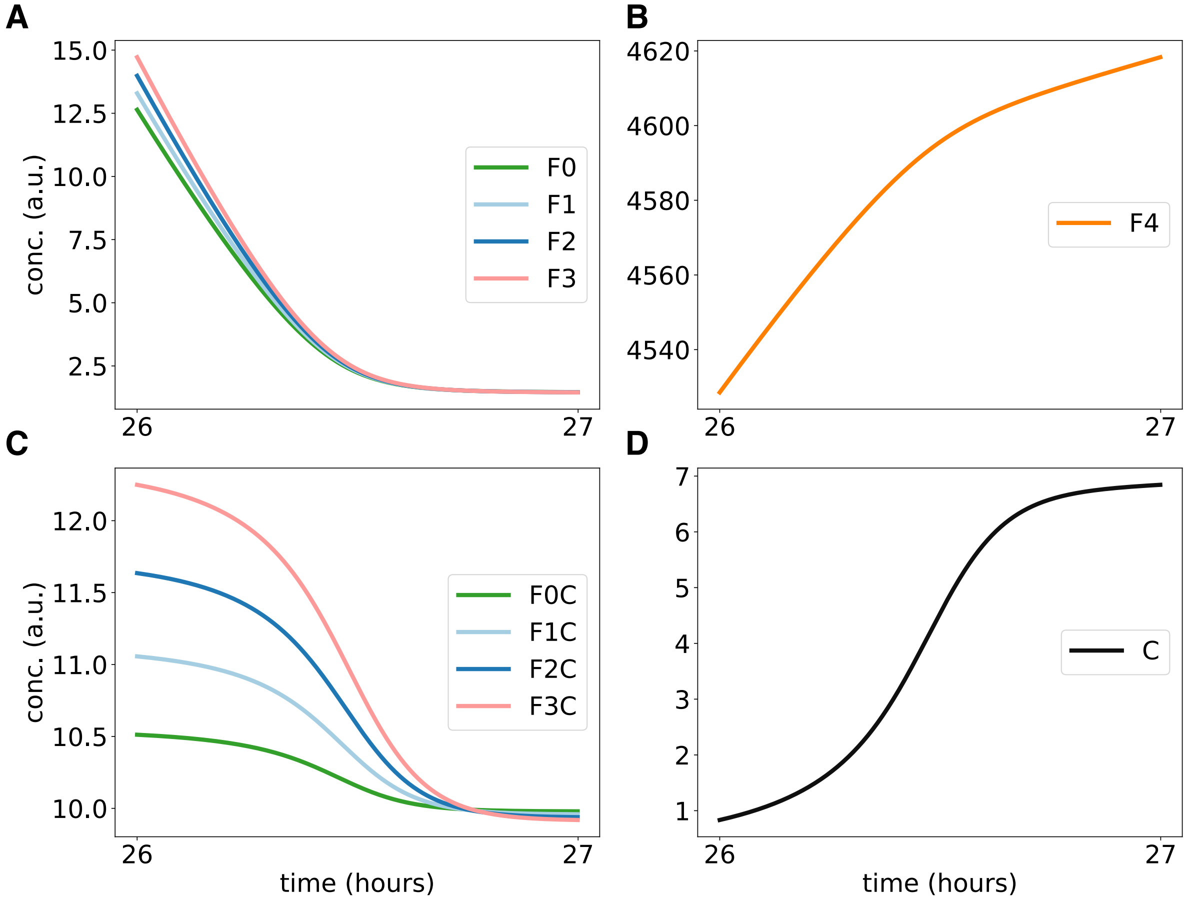

Figure A6. Magnified simulations of nonlinear phosphorylations from Fig 4: Graphs show a one hour time window (26 to 27 hours) and confirm that apparent kinks in Fig 4 are rather smooth curves. The asymptotic equilibrium indicates that most of the F protein is sequestered within complexes.

Appendix A.7 Effects of model extensions

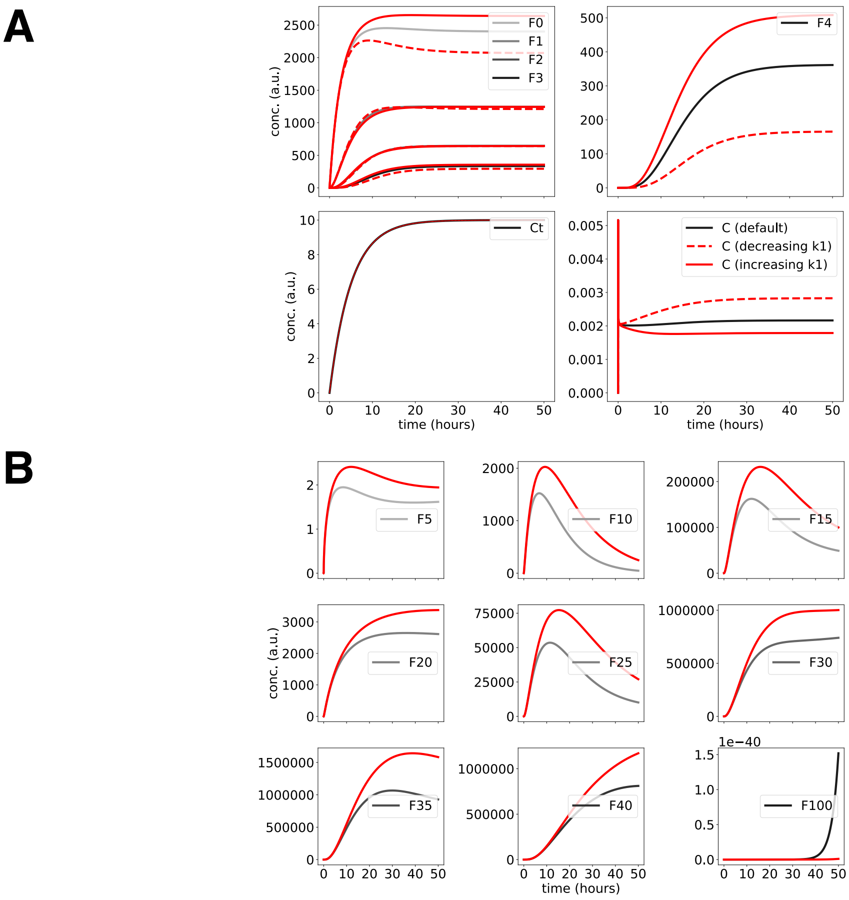

Figure A7. (A) Simulating decreasing and increasing phosphorylation rates: We compare constant phosphorylation rates k_1 (see Fig 3 and A2) with decreasing rates: 50, 40, 30, 20 and increasing rates: 50, 60, 70, 80 for increasing phosphorylation. These step by step changes of the rates mimic negative and positive cooperativity, respectively. The modifications have only minor effects on amplitudes, delays, and waveforms. **(B) Simulating stabilizing and destabilizing phosphorylations:** Compared to constant degradation rates k_d=1.5h^{-1} (grey curves) we take into account the stabilizing effects of initial phosphorylation and the destabilization of later phosphorylations. We decrease for k=0, 1,..., 50 k_d in every step by a factor 0.95 and increase k_d stepwise for k=51, 52,..., 100 by 0.95^{-1}. This modification leads to even higher amplitudes of intermediate phosphorylation levels (red curves).

353

354 ### *Appendix A.8 Multiple random phosphorylations in the circadian clock*

355 We modelled the nonlinear random phosphorylations in a circadian clock using a 9 variable ODE system
356 of n=4 phosphorylations and a Goodwin oscillator with 3 variables. Circadian rhythms and multiple random
357 phosphorylations are shown in Fig 9. Parameters in Fig 9: a_1=100h^{-1}, a_2=0.5h^{-1}, b_1=100h^{-1}, b_2=0.5h^{-1},
358 c_1=100h^{-1}, c_2=0.5h^{-1}, P_p=10000, k_1=1000, k_2=1000h^{-1} and k_d=15000h^{-1}.

A

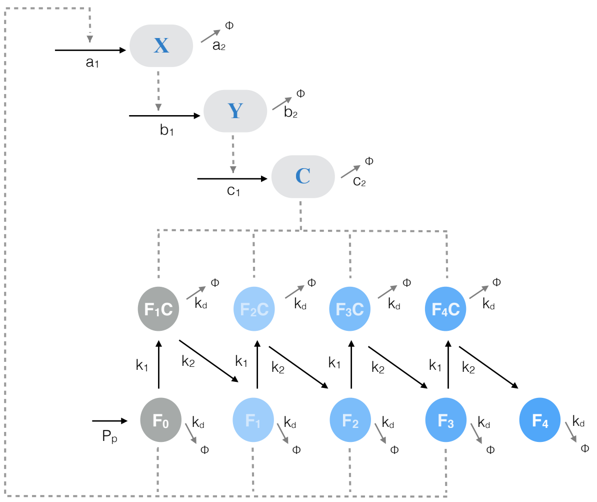

B

$$Fact = F0 + F1 + F2 + F3$$

$$\frac{d[X]}{dt} = a1 * Fact - a2 * X$$

$$\frac{d[Y]}{dt} = b1 * X - b2 * Y$$

$$\frac{d[C]}{dt} = c1 * Y - c2 * C + (k_2 * [F_1C] + k_2 * [F_2C] + k_2 * [F_3C] + k_2 * [F_4C]) - (k_1 * [F_0] * [C] + k_1 * [F_1] * [C] + k_1 * [F_2] * [C] + k_1 * [F_3] * [C])$$

$$\frac{d[F_0]}{dt} = P_p - k_1 * [F_0] * [C] - k_d * [F_0]$$

$$\frac{d[F_1C]}{dt} = k_1 * [F_1] * [C] - k_2 * [F_1C] - k_d * [F_1C]$$

$$\frac{d[F_1]}{dt} = 4 * k_2 * [F_1C] - k_1 * [F_1] * [C] - k_d * [F_1]$$

$$\frac{d[F_2C]}{dt} = k_1 * [F_1] * [C] - k_2 * [F_2C] - k_d * [F_2C]$$

$$\frac{d[F_2]}{dt} = 3 * k_2 * [F_2C] - k_1 * [F_2] * [C] - k_d * [F_2]$$

$$\frac{d[F_3C]}{dt} = k_1 * [F_2] * [C] - k_2 * [F_3C] - k_d * [F_3C]$$

$$\frac{d[F_3]}{dt} = 2 * k_2 * [F_3C] - k_1 * [F_3] * [C] - k_d * [F_3]$$

$$\frac{d[F_4C]}{dt} = k_1 * [F_3] * [C] - k_2 * [F_4C] - k_d * [F_4C]$$

$$\frac{d[F_4]}{dt} = k_2 * [F_4C] - k_d * [F_4]$$

Figure A8. (A) Schematic diagram of the multiple random phosphorylation combined with the Goodwin model: In the model, X represents the mRNA (e.g., frq mRNA), Y the protein (e.g., FRQ), and C the nuclear inhibitor complex (e.g., FFC) including kinase C. Protein F is phosphorylated by kinase C in a distributive random manner up to N=4 phosphorylation sites. **(B) Fusion model with up to n=4 random phosphorylations.**

Appendix A.9 Effects of constitutive kinase expression

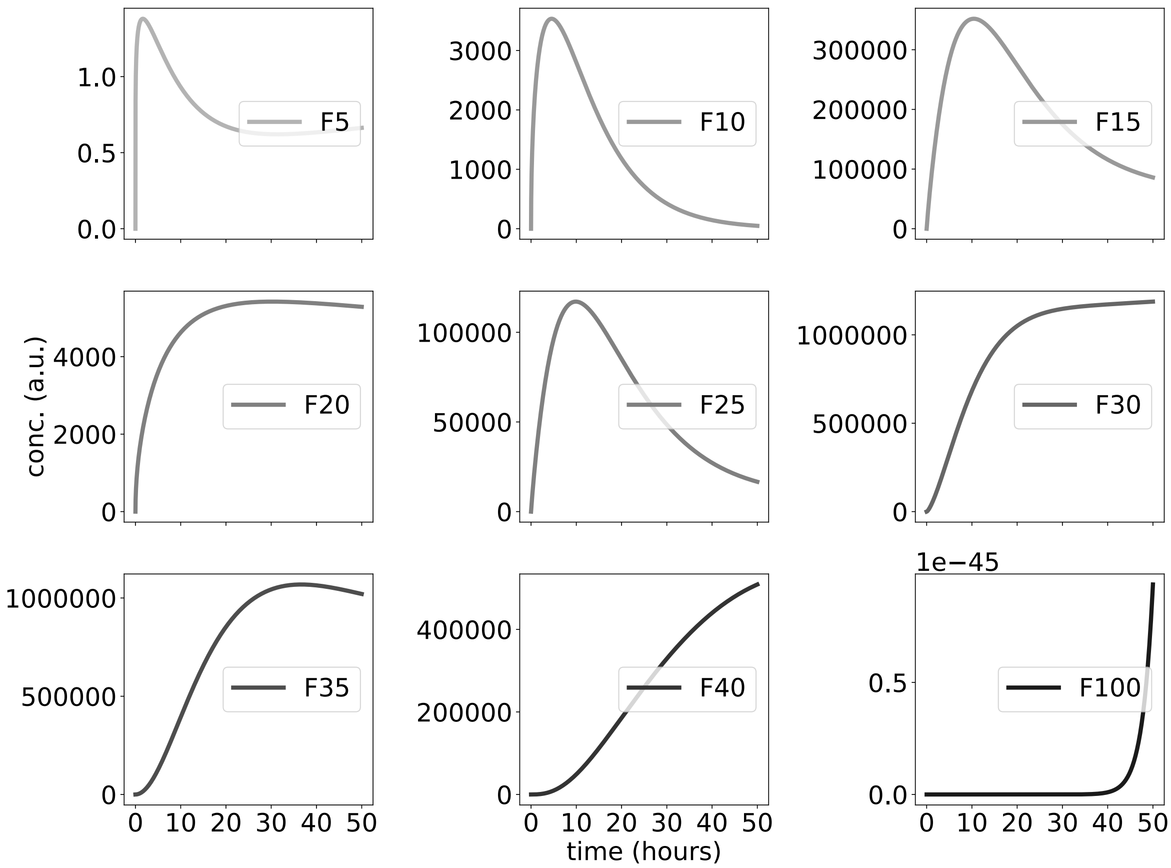

Figure A9. Simulating large scale nonlinear random phosphorylations using steady state level of total kinase: Graphs show almost similar time-courses of hypo, medium, hyperphosphorylated and fully phosphorylated F proteins for up to n=100 phosphorylations (compare Figure 7).
